# Cyclic GMP-AMP Displays Mucosal Adjuvant Activity in Mice

**DOI:** 10.1371/journal.pone.0110150

**Published:** 2014-10-08

**Authors:** Ivana Škrnjug, Carlos Alberto Guzmán, Christine Ruecker

**Affiliations:** Department of Vaccinology and Applied Microbiology, Helmholtz Centre for Infection Research, Braunschweig, Germany; The Ohio State University, United States of America

## Abstract

The recently discovered mammalian enzyme cyclic GMP-AMP synthase produces cyclic GMP-AMP (cGAMP) after being activated by pathogen-derived cytosolic double stranded DNA. The product can stimulate STING-dependent interferon type I signaling. Here, we explore the efficacy of cGAMP as a mucosal adjuvant in mice. We show that cGAMP can enhance the adaptive immune response to the model antigen ovalbumin. It promotes antigen specific IgG and a balanced Th1/Th2 lymphocyte response in immunized mice. A characteristic of the cGAMP-induced immune response is the slightly reduced induction of interleukin-17 as a hallmark of Th17 activity – a distinct feature that is not observed with other cyclic di-nucleotide adjuvants. We further characterize the innate immune stimulation activity *in vitro* on murine bone marrow-derived dendritic cells and human dendritic cells. The observed results suggest the consideration of cGAMP as a candidate mucosal adjuvant for human vaccines.

## Introduction

Cyclic di-nucleotides (CDNs) are bacterial second messengers with functions in motility and development. Bis-(3′,5′)-cyclic dimeric guanosine monophosphate (c-di-GMP), a member of this molecule family, is produced for example by the bacterium *Pseudomonas aeruginosa* in which it functions in biofilm formation [1], [2]. It was also described to be synthesized by the eukaryotic organism dictyostelium with implications in the regulation of motility and proliferation [3]. The related compound bis-(3′,5′)-cyclic dimeric adenosine monophosphate (c-di-AMP) is involved in the sporulation control of *Bacillus subtilis* [4] and has host immune modulatory activity in *Listeria monocytogenes* infection [5], [6]. Immunization experiments on mice using CDNs as adjuvants suggest that they can have the immune activity of pathogen associated molecular patterns (PAMPs). C-di-GMP, c-di-AMP, and the non-natural bis-(3′,5′)-cyclic dimeric inosine monophosphate were shown by us and others to have immune stimulatory effects and to promote balanced specific humoral and cellular responses upon immunization of mice [7]–[10].

The recently discovered mammalian enzyme cyclic GMP-AMP synthase (cGAS) synthesizes the CDN cyclic GMP-AMP (cGAMP) upon activation by foreign double stranded DNA [11]–[14]. It was proposed that cGAMP could serve as an adjuvant in vaccine formulations, because of its capability to activate innate immune responses [14]–[17]. Short term parenteral immunization studies with mice using the mammalian cGAS product c[G(2′,5′)pA(3′,5′)p] as adjuvant demonstrated enhanced antigen-specific immunoglobulin (Ig) G1 and T cell activity [15]. This cGAMP isomer was shown to bind the stimulator of interferon genes (STING) and activate interferon (IFN) type I production [13], [16], [18]. It was reported to have a higher affinity and more efficient activation potential toward human STING than the cGAMP isomer c[G(3′,5′)pA(3′,5′)p] that is produced by prokaryotes [11], [17], [19]. CGAMP would not qualify as a classical PAMP, since it can be produced by the host organism itself.

Here, we studied the effect of the prokaryotic cGAMP isomer (c[G(3′,5′)pA(3′,5′)p]) on the immune response to the model antigen ovalbumin (OVA) in mice. We chose the intra-nasal (i. n.) route, because we are especially interested in developing mucosal vaccines which were shown to evoke robust mucosal on top of systemic immunity [20], [21]. This feature makes mucosal vaccination more favorable in combating pathogens entering via mucosal surfaces of the host. We demonstrate the *in vivo* adjuvant effects of cGAMP on model antigen-specific humoral and cellular immune responses in mice. We further show that cGAMP can also induce the surface expression of select activation markers on human dendritic cells (DCs) *in vitro*.

## Materials and Methods

### Ethics statement

All animal experiments have been performed in agreement with the local government of Lower Saxony, Germany (approved permission No. 33.9-42502-04-12/0942).

Human blood was donated by healthy individuals who provided their written informed consent. The written consent’s documentation and archival storage procedures are approved by the regional Ethics Committee of Lower Saxony, Germany. The blood donors’ health is rigorously checked before being admitted for blood donation. This process includes a national standardized questionnaire with health questions, an interview with a medical doctor and standardized laboratory tests for HIV1/2, HBV, HCV, Syphilis infections and hematological cell counts. The blood donations were obtained from the Institute for Clinical Transfusion Medicine, Klinikum Braunschweig, Germany, in accordance with the declaration of Helsinki (2013) and approved by the regional Ethics Committee of Lower Saxony, Germany, and were analyzed anonymously.

### Mice

Female C57BL/6 (H-2b) mice 6–8 weeks old (Harlan, Rossdorf, Germany) were kept at the animal facility of the Helmholtz Centre for Infection Research under specific pathogen-free conditions. Animals were randomly assigned to experimental groups.

### Cyclic di-nucleotides

Lyophilized cGAMP c[G(3′,5′)pA(3′,5′)p] (InvivoGen, San Diego, California, USA; BioLog, Bremen, Germany) and c-di-AMP (BioLog, Bremen, Germany) were dissolved in water (Ampuwa; Serumwerk, Bernburg, Germany). LPS contamination higher than 1.6 ng/ml was ruled out by employing the HEK-Blue LPS Detection Kit (InvivoGen, San Diego, California, USA) (Figure S1).

### Preparation of murine bone marrow-derived cells

The femur and tibia from mice were flushed with medium (RPMI 1640, 10% v/v fetal calf serum (FCS), 100 U/ml penicillin, 50 µg/ml streptomycin, 100 µg/ml gentamycin; Gibco, USA). Erythrocytes were lysed in 150 mM NH_4_Cl, 10 mM KHCO_3_, 0.1 mM EDTA (Sigma-Aldrich, Steinheim, Germany), pH 7.2. 10^6^ cells/ml were seeded in medium with 5 ng/ml murine granulocyte macrophage colony-stimulating factor (GM-CSF) (BD Pharmingen, USA) and cultured for 7 days at 37°C.

### Preparation of human DCs from peripheral blood mononuclear cells (PBMCs)

PBMCs were isolated from human blood donations by centrifugation on Ficoll (GE Healthcare, Sweden). DCs were prepared using the Myeloid DC Isolation Kit, human (Miltenyi, Germany) and recovered overnight in medium (RPMI 1640, 10% v/v FCS, 100 U/ml penicillin, 100 µg/ml streptomycin; Gibco) at 37°C.

### *In vitro* stimulation of primary cells

The culture medium of primary cells was supplemented with 5 µg/ml (murine cells) or 60 µg/ml (human cells) of c-di-AMP or cGAMP or left without additive. Cells were incubated for 24 h at 37°C.

### Flow cytometric analysis of *in vitro*-stimulated primary cells

Cells were collected in phosphate buffered saline (PBS) and pre-incubated with Fc receptor blocking anti-mouse CD16/CD32 (clone 93) or human Fc receptor binding inhibitor (eBioscience Inc., USA). A blue fluorescent amine-reactive dye (Invitrogen, USA) was used as live/dead cell marker. Murine cells were decorated with anti-mouse CD80 (clone 16-10A1, APC-conjugated), CD86 (clone GL1, PE-conjugated), I-A^b^ (clone AF6-120.1, FITC-conjugated), CD11c (clone N418, PE-Cy7-conjugated); human cells with anti-human CD40 (clone 5C3, PE-conjugated), CD54 (clone HA58, APC-conjugated), CD80 (clone 2D10, APC-conjugated), CD83 (clone HB15e, PE-conjugated), CD86 (clone IT2.2, PE-Cy7-conjugated), CD11c (clone 3.9, Brilliant Violet 711-conjugated) (BioLegend, USA). Flow cytometry analysis was performed using an LSR-II and the software FACSDiva (BD Bioscience, USA) and FlowJo Mac v9.6 (Tree Star, Inc., USA). Forward and sideward scatter gating was used to restrict fluorescence analysis to intact single cells only. Live cells were gated based on their lower blue fluorescence emission of the live/dead cell marker and DCs were identified by their enhanced surface expression of CD11c. Activated cells were identified by gating for cell populations with enhanced fluorescence intensity indicative of the antibody-detected surface molecules.

### Flow cytometric analysis of intracellular IL-17 in re-stimulated spleen cells

Spleen cells from immunized mice were incubated for 16 h at 37°C in the presence of 40 µg/ml OVA. Then, 5 µg/ml brefeldin A and 6 µg/ml monensin were added and the cells were incubated for another 8 h at 37°C. Cells were collected in PBS and stained with live/dead cell marker (see above) and decorated with the following fluorophore-conjugated antibodies: anti-mouse CD3 (clone: 145-2c11, FITC-conjugated), CD4 (clone: RM4-5, PE-Cy7-conjugated) (eBioscience Inc., USA). Cells were fixed in 1% paraformaldehyde in PBS, washed, permeabilized in 0.5% w/v BSA (Roth, Karlsruhe, Germany), 0.5% w/v saponin (Serva, Heidelberg, Germany) in PBS and decorated with anti-mouse IL-17 (clone TC11-1 8H10, V450-conjugated) (BD Bioscience, USA). Live CD3^+^/CD4^+^ cells were gated and analyzed for IL-17^+^ cells (see information above on flow cytometer and analysis software).

### Mouse immunization experiments

Five animals per group were immunized i. n. on days 0, 14 and 28. Animals were anesthetized with Isoflurane (Abbott Animal Health, USA) and treated 10 µl per nostril with 15 µg OVA (EndoGrade, Hyglos, Germany) (Figure S1) alone or co-administered with 5 µg per dose of c-di-AMP, cGAMP or cholera toxin B subunit (CTB; Sigma-Aldrich) (Figure S1) in Ampuwa or with Ampuwa alone in the control group (mock immunization). On day 42 after immunization animals were sacrificed and samples were collected.

### Sample collection

Blood samples were collected from the retro-orbital complex on days -1, 13 and 42, centrifuged at 8000×g, 5 min, and sera were stored at −20°C. Spleens were isolated, pooled per groups, transferred to medium (RPMI 164, 10% v/v FCS, 100 U/ml penicillin, 50 µg/ml streptomycin, 100 µg/ml gentamycin and 2-mercaptoethanol 50 µM; Gibco), and gently pressed through a 100 µm cell mesh. Erythrocytes were lysed in 150 mM NH_4_Cl, 10 mM KHCO_3_, 0.1 mM EDTA (pH 7.2) and cells were washed. To isolate cells from the cervical lymph nodes (CLN) lymph nodes were also pooled per groups, transferred to medium (see above), pressed through a 100 µm pore nylon mesh and washed. Nasal lavage samples were collected by flushing the nasal cavity and stored at −20°C in 300 µl PBS supplemented with FCS (5% v/v) and 2 mM PMSF (Sigma-Aldrich).

### Spleen cell proliferation assay

Spleen cells (4×10^5^) were stimulated with 4 µg/ml OVA, incubated for 72 h at 37°C, then incubated for another 16 h in the presence of 20 µCi/ml ^3^H-thymidine (PerkinElmer, USA) and harvested on Filtermat A filters (PerkinElmer, USA) using cell harvester (Inotech, Switzerland). Incorporated ^3^H-thymidine was measured by a γ scintillation counter (1450 Microbeta Trilux, Wallach Sverige, Sweden). The blank values were subtracted from values obtained with re-stimulated cells and the average value for each group was calculated.

### Measurement of IgG titers in the serum of immunized mice by ELISA

OVA-specific antibodies in serum samples were measured by IgG total or isotype specific ELISA. The plates were coated overnight at 4°C with OVA (2 µg/ml in 0.05 M carbonate buffer, pH 8.2) and blocked with 3% w/v bovine serum albumin (BSA) in PBS for 1 h at 37°C. Serial 2-fold dilutions of the samples were prepared in 3% w/v BSA in PBS and incubated for 2 h at 37°C, washed 6 times with 0.1% v/v Tween20 in PBS. The biotinylated detection antibodies goat, anti-mouse IgG (Sigma-Aldrich), goat anti-mouse IgG1 or goat, anti-mouse IgG2c (Southern biotech, USA), 1∶5000 in 1% w/v BSA/0.1% v/v Tween20 in PBS, were used for incubation for 2 h at 37°C. Six washes were followed by incubation with Streptavidin-horse radish peroxidase (HRP) (BD Pharmingen), 1∶1000 in 1% w/v BSA/0.1% v/v Tween20 in PBS, for 1 h at 37°C, six washes, incubation with ABTS [2,20-azino-bis(3-ethylbenzthiazoline-6-sulfonic acid)] in 0.1 M citrate-phosphate buffer (pH 4.35) and 0.03% H_2_O_2_ (Sigma-Aldrich), and the absorbance of light at 405 nm wave length was measured using a Synergy 2 Multi-Mode Microplate Reader (Biotek, USA). Titers were determined as the highest dilution factors of the assay samples with twice the average readout value of the blank.

### Measurement of IgA titers in the nasal lavage of immunized mice by ELISA

The OVA-specific IgA response was determined in ELISA with OVA-coated plates, total IgA was determined in ELISA with anti-mouse IgA (Southern biotech, USA)-coated plates for both using anti-mouse IgA biotinylated goat, anti-mouse IgA (Southern biotech, USA) for detection [22] following the procedures described for measuring OVA-specific IgG titers. The titers of total IgA and of OVA-specific IgA were determined as the highest dilution factors of the assay samples with twice the average readout value of the blank. For normalization, the percentage of OVA-specific IgA titer of the total IgA titer was calculated for each sample.

### Detection of cytokine production in spleen cells from immunized mice by ELISPOT analysis

The numbers of IFN-γ, interleukin (IL)-2, IL-4 and IL-17 producing cells from the spleen or the CLN were analyzed using MultiScreen HTS filter plates (Millipore, Germany) and mouse ELISPOT pair antibodies (BD Bioscience) according to the manufactureŕs instructions. Spleen cells (4×10^5^) were incubated for 24 h (IFN-γ) or 48 h (IL-2, IL-4, IL-17) in the presence of 30 µg/ml OVA at 37°C. A CTL ELISPOT reader and the ImmunoSpot image analyzer software v3.2 (Cellular Technology, Ltd., Germany) were used. The values of cells without re-stimulation were subtracted from those obtained with OVA re-stimulated cells and the average value for each of the groups was calculated. The subtracted background was always below five percent of the spot number value for the samples from immunization experiments with CDN-adjuvanted OVA.

### Statistical analysis

The statistical analysis was performed by applying the unpaired t-test; n.s. indicates not significant; ***indicates p<0.001; **indicates p<0.01; and *indicates p<0.05.

## Results

To characterize the potential immune enhancing effects of cGAMP we designed immunization experiments comparing immune responses to an antigen with and without cGAMP supplementation. The experiments included five groups of five mice each: the first group received the model antigen OVA alone, the second group received OVA in combination with cGAMP and as controls the third group received OVA with the standard adjuvant CTB, the fourth group received OVA in combination with the model cyclic di-nucleotide c-di-AMP and the fifth group received neither the antigen OVA nor any other supplement (the mock control). The different formulations were applied i. n., the mice were boosted with the respective formulations two weeks and four weeks after the first immunization and were sacrificed six weeks after the first application. Samples were analyzed for parameters of immune response characterization.

### cGAMP promotes antigen-specific cellular immune responses in immunized mice

**Figure 1:**
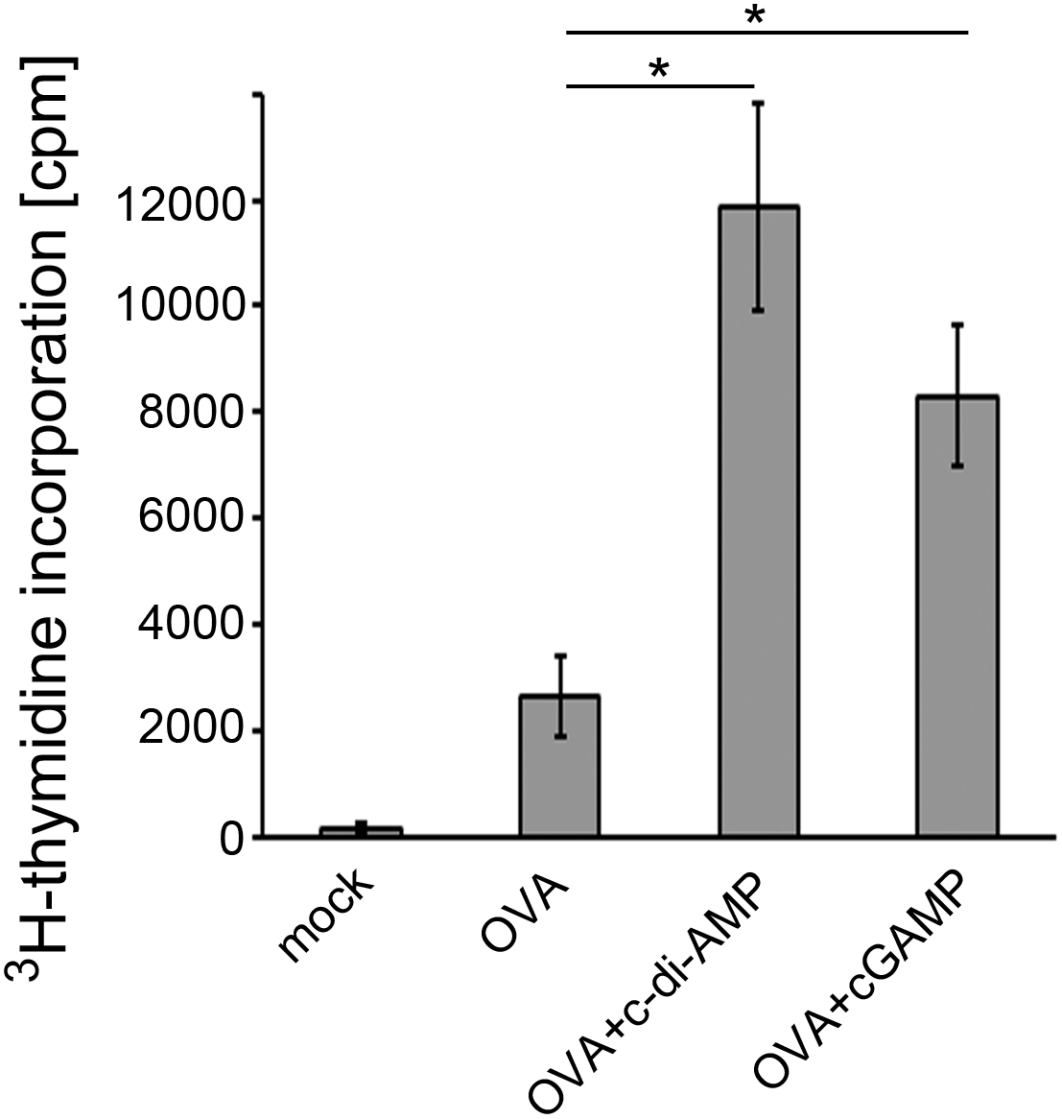
cGAMP promotes the antigen-specific proliferation capacity of spleen cells in mice. Proliferation of antigen-stimulated spleen cells from mice immunized with the model antigen OVA alone or adjuvanted with the model cyclic di-nucleotide c-di-AMP or cGAMP was measured by ^3^H-thymidine incorporation. Spleen cells of the mock sample were taken from antigen and adjuvant free immunization control mice. The error bars represent the SEM of three independent experiments with five mice per group per experiment. Significant differences between select groups are indicated with * for p<0.05.

First, spleen cells were isolated and proliferation upon *in vitro* re-stimulation with OVA was assessed by ^3^H-thymidine incorporation. Spleen cells from mice immunized with the antigen OVA alone responded with proliferation to the presence of OVA. However, co-administration of the model cyclic di-nucleotide c-di-AMPand cGAMP resulted in enhanced proliferative capacity of the re-stimulated cells (Figure 1).

**Figure 2:**
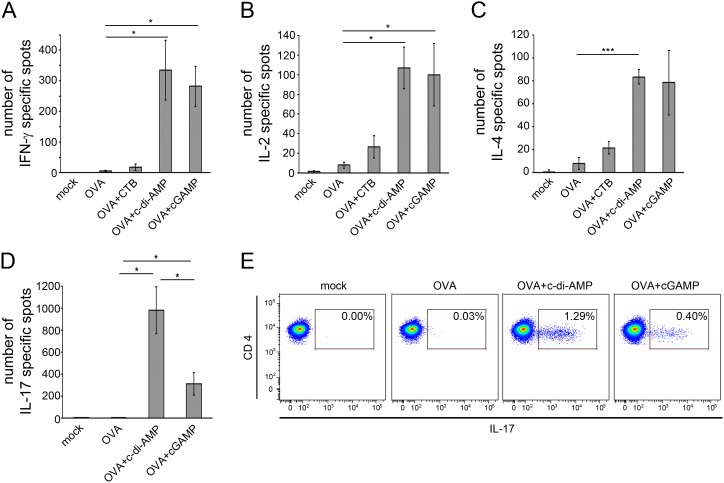
cGAMP promotes the antigen-specific cytokine production by spleen cells of immunized mice. Mice were immunized with OVA alone or adjuvanted with CTB, c-di-AMP or cGAMP. Spleen cells of these mice were re-stimulated with OVA and analyzed for the production of the cytokines (**A**) IFN-γ, (**B**) IL-2, (**C**) IL-4 and (**D**) IL-17 in ELISPOT assays. The number of spots is given for 10^6^ cells. The error bars represent the SEM of three independent experiments with five mice per group per experiment. Significant differences between select groups are indicated with * for p<0.05 and *** for p<0.001. (**E**) IL-17 producing cells were detected by flow cytometry analysis. Shown are the fluorescence signals on dot plots of cells with intracellular presence of IL-17 as percentage of 95700 gated CD3^+^/CD4^+^ cells.

Second, we sought to identify the cell types that were specifically activated by OVA. To this end, we applied ELISPOT assays measuring the production of T helper (Th) lymphocyte type indicator cytokines, such as IFN-γ and IL-2 for Th1 cells, IL-4 for Th2 cells, and IL-17 for Th17 cells [23], [24]. A very pronounced enhancement in numbers of IFN-γ and IL-2 secreting cells was observed for the samples derived from mice immunized with OVA in combination with c-di-AMP or cGAMP when compared to samples from immunizations with the antigen OVA alone (Figure 2). A quite similar observation was made for IL-4 and IL-17 producing cells (Figure 2). However, the number of IL-17 producing cells was much higher in the samples from mice immunized with the OVA/c-di-AMP combination than for those immunized with OVA/cGAMP (Figure 2D). Taken together these results suggest that cGAMP has the potential to promote a balanced specific immune response with regard to Th cell types. This response lies in the same range as that evoked in the presence of c-di-AMP, albeit with a less pronounced but still facilitated Th17 response when compared to immunizations with non-supplemented antigen. We further substantiated the differential IL-17 production in response to c-di-AMP *vs* cGAMP adjuvanted antigen by flow cytometry analysis of intracellular IL-17 expression in CD3^+^/CD4^+^ cells (Figure 2E). The obtained results confirm that cGAMP enhances the IL-17 production in comparison to OVA alone, but to a lesser extent than c-di-AMP.

### cGAMP promotes antigen-specific humoral immune responses in immunized mice

**Figure 3:**
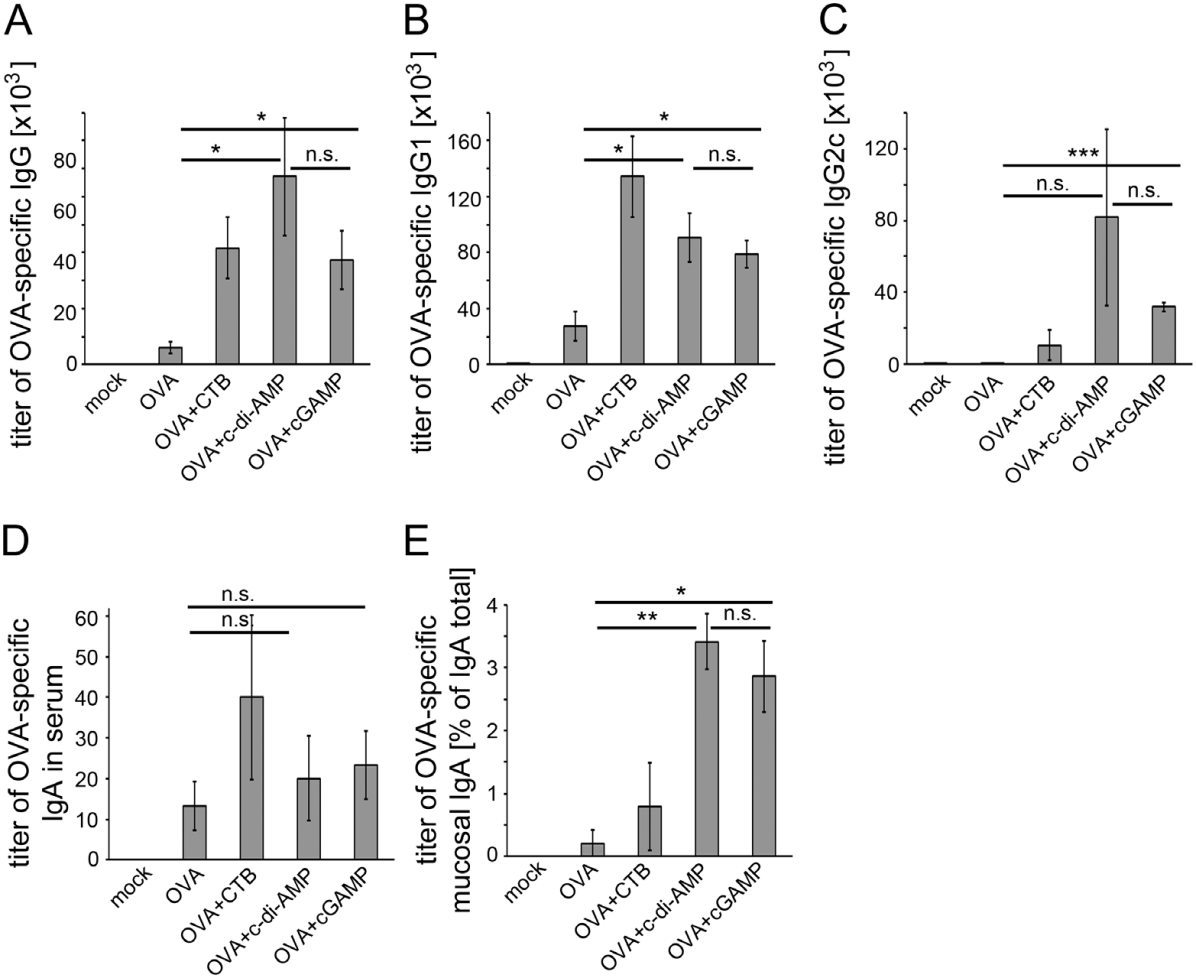
cGAMP promotes antigen-specific Ig responses in immunized mice. Mice were immunized with OVA alone or adjuvanted with CTB, c-di-AMP or cGAMP. Sera samples of these mice were analyzed for their OVA-specific (**A**) total IgG, (**B**) IgG1, (**C**) IgG2c, (**D**) IgA titers by ELISA. The titer gives the dilution factor of the assay sample with twice the readout value of the blank (absorbance of light of 405 nm wave length). (**E**) Mucosal IgA was analyzed in nasal lavage samples by ELISA and is given as percentage of OVA-specific IgA of total IgA titers. The error bars represent the SEM of three independent experiments with five mice per group per experiment. Significant differences between select groups are indicated with * for p<0.05 and ** for p<0.01.

Next we asked if cGAMP can also enhance the specific humoral response to an antigen when used as its adjuvant. We analyzed the sera of the immunized mice described above with regard to their content of OVA-specific IgG and IgA by ELISA. We found higher titers of OVA-specific IgA and total IgG as well as IgG1 and IgG2c in the sera of mice immunized with cGAMP-adjuvanted OVA as compared to sera from OVA-immunized mice (Figure 3A–D). The elevated titers of IgG1 and IgG2c also point to an enhanced activity of Th2 and Th1 cells, respectively [25]. Thus, we found the suggested balanced Th1/Th2 cell response based on the cytokine production (Figure 2A–C) confirmed by the results presented in figure 3. Nasal lavage was performed on the immunized mice to obtain samples for the analysis of mucosal OVA-specific IgA responses upon supplementary cGAMP administration. cGAMP indeed enhanced the mucosal antigen-specific IgA titers as compared to samples from mice immunized with OVA without cGAMP (Figure 3E).

### cGAMP directly activates murine and human dendritic cells *in vitro*

**Figure 4:**
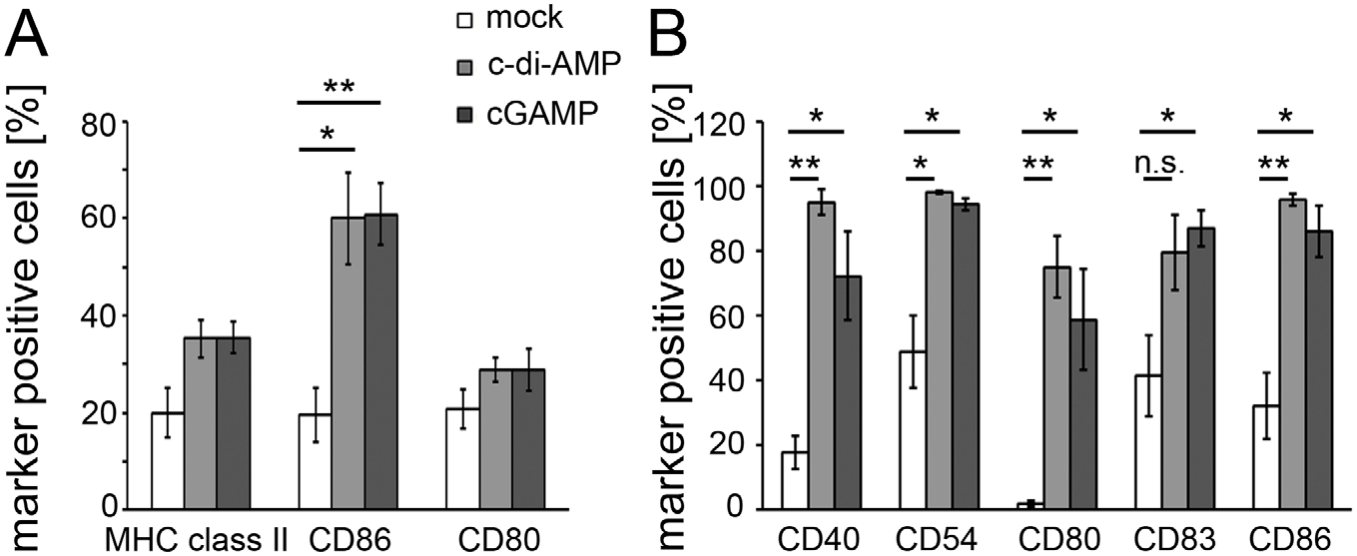
cGAMP up-regulates surface expression of activation markers on murine and human dendritic cells. (**A**) Murine bone marrow-derived DCs and (**B**) human PBMC-derived DCs were stimulated *in vitro* with c-di-AMP, cGAMP (both at (**A**) 5 µg/ml or (**B**) 60 µg/ml) or left untreated (mock) for 24 h. The DCs were decorated with fluorophore-conjugated antibodies against the DC activation markers CD40, CD54, CD80, CD83, CD86 or MHC class II (I-A^b^) and analyzed by flow cytometry. Shown are the percentages of marker-positive CD11c^+^ cells of the three independent experiments. Error bars are SEM of three independent experiments.

We tested the capacity of cGAMP to activate murine innate immune cells to establish an *in vitro* readout that can also be applied to human immune cells. Such experiments would allow first conclusions on the potential of cGAMP to be used as an adjuvant in human vaccines. Hence, cGAMP was added to the culture medium of murine bone marrow-derived DCs (cultured in the presence of GM-CSF) and the surface expression of the activation markers CD80, CD86 and MHC class II was assessed by flow cytometry (Figures 4A and S3B). The observed up-regulation of the activation markers on cGAMP- and c-di-AMP-stimulated DCs in comparison to the non-stimulated mock control DCs suggests that cGAMP directly activates murine innate immune cells with a capacity resembling that of the model CDN c-di-AMP. Similarly, cGAMP can also activate human PBMC-derived DCs as indicated by the up-regulation of the human DC activation markers CD40, CD54, CD80, CD83 and CD86 (Figures 4B and S3A). The purity of the tested cell populations with respect to CD11c^+^ cells was 95% for the human PBMC-derived DCs and 82% for the mouse bone marrow-derived DCs. Hence, we concluded that the observed activation of the DCs was predominantly due to direct effects of c-di-AMP and cGAMP on these cells rather than mediated by CD11c^−^ bystander cells present in the preparations.

## Discussion

The formulation of modern subunit vaccines usually requires the addition of adjuvants to compensate for the rather low immunogenicity of isolated antigens. Not many adjuvants are approved for the use in human vaccines. Especially for mucosal vaccination, for example via the nasal route, adjuvants need to be suited to cross the mucosal barrier but at the same time have to meet high safety standards. Molecules such as cGAMP that are produced by the mammalian organism itself are expected to be of very low toxicity. Here we demonstrate the efficacy of cGAMP as an i. n. administered mucosal adjuvant in a pre-clinical mouse model. The cGAMP-dependent enhanced specific IgG and IgA titers in our mouse immunization experiments (Figure 3) suggest that the use of cGAMP promotes the antigen-specific humoral immune response. Spleen cells from mice immunized with cGAMP-adjuvanted antigen showed a facilitated antigen-specific proliferation capacity (Figure 1). They secrete cytokines indicating antigen-specific activity of different Th cell types (Figure 2). A similar secretion profile was observed in cells obtained from the CLN of immunized mice (Figure S2). The analysis of the cytokines produced by re-stimulated cells obtained from immunized mice six weeks after the last boost provides a first indication of the potential of cGAMP to trigger long-term immunity (Figure S4). These observations demonstrate the efficacy of cGAMP as an adjuvant that promotes a broad cellular immune response in our mouse immunization model. The induction of considerable Th1 cell activity and of IL-17 secretion at a comparatively low level mediated by cGAMP could be a valuable unique feature for the development of vaccines with defined specific effects. Different pathogens require different immune responses to be controlled: for example, a pronounced Th1 activity is needed to combat intracellular pathogens while a robust Th2 stimulation aids the immune response against extracellular bacteria or parasites. Both activities were promoted by cGAMP. Notably, the use of cGAMP as an adjuvant in our mouse immunization model gave rise to a very robustly enhanced number of IFN-γ and IL-2 secreting cells (Figure 2) suggesting a considerable Th1 response. This can hardly be achieved with the use of alum as a widespread adjuvant among vaccines currently approved for humans [26], [27]. IL-17 signaling, largely provided by activated Th17 cells, can also modulate Th1 responses [28]. In the here described immune response to OVA/cGAMP, the observed Th1 indicator molecule profile resembled that induced in the OVA/c-di-AMP immunized mice (Figure 2). This suggests that Th1 profiles are not strongly dependent on IL-17 modulation. However, it was also reported that IL-17 activity can have adverse effects. For example, vaccinated mice can develop arthritis after the challenge with *Borrelia burgdorferi* in an IL-17 dependent manner [29]. In such cases one may want to compose vaccines that provide control over IL-17 secretion activity, for instance by using cGAMP as an adjuvant.

The observed *in vitro* responsiveness of human innate immune cells (Figure 4B) indicates a promising activity of cGAMP in the human background. It suggests that cGAMP does not exclusively act on mouse-specific sensor molecules to cause immune cell activation and qualifies cGAMP to be considered as candidate adjuvant for the use in human vaccines. One pathway that was suggested to be activated by cGAMP is the STING-mediated IFN type I production. It was reported that STING variants of different species show differential binding or activation behavior with the canonical cGAMP isomer c[G(3′,5′)pA(3′,5′)p] and the non-canonical isomer c[G(2′,5′)pA(3′,5′)p] which is the mammalian cGAS product. As opposed to murine STING, human STING was reported to prefer [12] or exclusively respond to [11], [17] the non-canonical c[G(2′,5′)pA(3′,5′)p] isomer of cGAMP. Here, we demonstrate the activity of the canonical c[G(3′,5′)pA(3′,5′)p] on human innate immune cells.

Taken together, the activity of cGAMP as a mucosal adjuvant promotes model antigen specific adaptive immune responses in a pre-clinical model. The distinct profile of cGAMP adjuvant effects, especially with regard to its Th1/Th2/Th17 induction, makes it an interesting candidate adjuvant with predictable immune modulation properties and, hence, a valuable tool with a potential in rational vaccine design [30].

## Supporting Information

Figure S1 **Test of reagents used in immunization experiments for endotoxin activity.** Potential LPS contamination of the model antigen OVA and the adjuvants CTB, c-di-AMP and cGAMP was tested by employing the HEK-Blue LPS Detection Kit (Invivogen, USA) according to the manufacturer’s instructions. The substances were applied in the medium of the HEK-Blue cultures at the following concentrations: LPS with 1.625 ng/ml, OVA with 15 µg/ml, CTB with 25 µg/ml, c-di-AMP and cGAMP with 25 µg/ml. LPS was clearly detected by the readout absorbance whereas the absorbance values of OVA, CTB, c-di-AMP and cGAMP are at the same level as the blank value. The values represent duplicates, the error bars are SD.
(TIF)

Figure S2 **cGAMP-promotes antigen-specific cytokine production by cells of the spleen and the cervical lymph nodes (CLN).** Mice were immunized with OVA alone or OVA adjuvanted with either c-di-AMP or cGAMP. Cells from the spleen and the CLN of these mice were re-stimulated with OVA and analyzed for the production of the cytokines (**A**) IFN-γ, (**B**) IL-2, (**C**) IL-4 and (**D**) IL-17 in ELISPOT assays. The number of spots is given for 10^6^ cells.
(TIF)

Figure S3 **cGAMP up-regulates the surface expression of activation markers on human and murine dendritic cells.** Human PBMC-derived DCs (**A**) and murine bone marrow-derived DCs (**B**) were stimulated *in vitro* with c-di-AMP, cGAMP or left untreated (mock) for 24 h. The DCs were decorated with fluorophore-conjugated antibodies against the markers CD40, CD54, CD80, CD83, CD86 and MHC class II (I-A^b^) and further analyzed by flow cytometry. Histograms for activation marker analysis on CD11c^+^ cells are shown for each single experiment, one row representing data of one experiment. The horizontal bars show the applied gates for marker-positive cells.
(TIF)

Figure S4 **cGAMP-promoted antigen-specific cytokine production by spleen cells persists for six weeks after the last immunization boost.** Mice were immunized with OVA alone or OVA adjuvanted with c-di-AMP or cGAMP. The mice were sacrificed six weeks after the last boost and spleen cells of these mice were re-stimulated with OVA and analyzed for the production of the cytokines (**A**) IFN-γ, (**B**) IL-2, (**C**) IL-4 and (**D**) IL-17 in ELISPOT assays. The number of spots is given for 10^6^ cells.
(TIF)

## References

[1] Karaolis DK, Rashid MH, Chythanya R, Luo W, Hyodo M, et al (2005) c-di-GMP (3′-5′-cyclic diguanylic acid) inhibits Staphylococcus aureus cell-cell interactions and biofilm formation. Antimicrob Agents Chemother 49: 1029–1038. 15728899 10.1128/AAC.49.3.1029-1038.2005 PMC549248

[2] Meissner A, Wild V, Simm R, Rohde M, Erck C, et al (2007) Pseudomonas aeruginosa cupA-encoded fimbriae expression is regulated by a GGDEF and EAL domain-dependent modulation of the intracellular level of cyclic diguanylate. Environ Microbiol 9: 2475–2485. 17803773 10.1111/j.1462-2920.2007.01366.x

[3] Chen ZH, Schaap P (2012) The prokaryote messenger c-di-GMP triggers stalk cell differentiation in Dictyostelium. Nature 488: 680–683. 22864416 10.1038/nature11313 PMC3939355

[4] Oppenheimer-Shaanan Y, Wexselblatt E, Katzhendler J, Yavin E, Ben-Yehuda S (2011) c-di-AMP reports DNA integrity during sporulation in Bacillus subtilis. EMBO Rep 12: 594–601. 21566650 10.1038/embor.2011.77 PMC3128283

[5] Woodward JJ, Iavarone AT, Portnoy DA (2010) c-di-AMP secreted by intracellular Listeria monocytogenes activates a host type I interferon response. Science 328: 1703–1705. 20508090 10.1126/science.1189801 PMC3156580

[6] Yamamoto T, Hara H, Tsuchiya K, Sakai S, Fang R, et al (2012) Listeria monocytogenes strain-specific impairment of the TetR regulator underlies the drastic increase in cyclic di-AMP secretion and beta interferon-inducing ability. Infect Immun 80: 2323–2332. 22508860 10.1128/IAI.06162-11 PMC3416470

[7] Ebensen T, Libanova R, Schulze K, Yevsa T, Morr M, et al (2011) Bis-(3′,5′)-cyclic dimeric adenosine monophosphate: strong Th1/Th2/Th17 promoting mucosal adjuvant. Vaccine 29: 5210–5220. 21619907 10.1016/j.vaccine.2011.05.026

[8] Libanova R, Becker PD, Guzman CA (2012) Cyclic di-nucleotides: new era for small molecules as adjuvants. Microb Biotechnol 5: 168–176. 21958423 10.1111/j.1751-7915.2011.00306.x PMC3815777

[9] Libanova R, Ebensen T, Schulze K, Bruhn D, Norder M, et al (2010) The member of the cyclic di-nucleotide family bis-(3′,5′)-cyclic dimeric inosine monophosphate exerts potent activity as mucosal adjuvant. Vaccine 28: 2249–2258. 20060510 10.1016/j.vaccine.2009.12.045

[10] Karaolis DK, Means TK, Yang D, Takahashi M, Yoshimura T, et al (2007) Bacterial c-di-GMP is an immunostimulatory molecule. J Immunol 178: 2171–2181. 17277122 10.4049/jimmunol.178.4.2171

[11] Ablasser A, Goldeck M, Cavlar T, Deimling T, Witte G, et al (2013) cGAS produces a 2′-5′-linked cyclic dinucleotide second messenger that activates STING. Nature 498: 380–384. 23722158 10.1038/nature12306 PMC4143541

[12] Gao P, Ascano M, Wu Y, Barchet W, Gaffney BL, et al (2013) Cyclic [G(2′,5′)pA(3′,5′)p] is the metazoan second messenger produced by DNA-activated cyclic GMP-AMP synthase. Cell 153: 1094–1107. 23647843 10.1016/j.cell.2013.04.046 PMC4382009

[13] Sun L, Wu J, Du F, Chen X, Chen ZJ (2013) Cyclic GMP-AMP synthase is a cytosolic DNA sensor that activates the type I interferon pathway. Science 339: 786–791. 23258413 10.1126/science.1232458 PMC3863629

[14] Wu J, Sun L, Chen X, Du F, Shi H, et al (2013) Cyclic GMP-AMP is an endogenous second messenger in innate immune signaling by cytosolic DNA. Science 339: 826–830. 23258412 10.1126/science.1229963 PMC3855410

[15] Li XD, Wu J, Gao D, Wang H, Sun L, et al (2013) Pivotal roles of cGAS-cGAMP signaling in antiviral defense and immune adjuvant effects. Science 341: 1390–1394. 23989956 10.1126/science.1244040 PMC3863637

[16] Gao D, Wu J, Wu YT, Du F, Aroh C, et al (2013) Cyclic GMP-AMP synthase is an innate immune sensor of HIV and other retroviruses. Science 341: 903–906. 23929945 10.1126/science.1240933 PMC3860819

[17] Diner EJ, Burdette DL, Wilson SC, Monroe KM, Kellenberger CA, et al (2013) The innate immune DNA sensor cGAS produces a noncanonical cyclic dinucleotide that activates human STING. Cell Rep 3: 1355–1361. 23707065 10.1016/j.celrep.2013.05.009 PMC3706192

[18] Gao P, Ascano M, Zillinger T, Wang W, Dai P, et al (2013) Structure-Function Analysis of STING Activation by c[G(2′,5′)pA(3′,5′)p] and Targeting by Antiviral DMXAA. Cell 154: 748–762. 23910378 10.1016/j.cell.2013.07.023 PMC4386733

[19] Zhang X, Shi H, Wu J, Sun L, Chen C, et al (2013) Cyclic GMP-AMP Containing Mixed Phosphodiester Linkages Is An Endogenous High-Affinity Ligand for STING. Mol Cell 51: 226–235. 23747010 10.1016/j.molcel.2013.05.022 PMC3808999

[20] Neutra MR, Kozlowski PA (2006) Mucosal vaccines: the promise and the challenge. Nat Rev Immunol 6: 148–158. 16491139 10.1038/nri1777

[21] Harandi AM, Medaglini D (2010) Mucosal adjuvants. Curr HIV Res 8: 330–335. 20353395 10.2174/157016210791208695

[22] Medina E, Paglia P, Rohde M, Colombo MP, Guzman CA (2000) Modulation of host immune responses stimulated by Salmonella vaccine carrier strains by using different promoters to drive the expression of the recombinant antigen. Eur J Immunol 30: 768–777. 10741391 10.1002/1521-4141(200003)30:3<768::AID-IMMU768>3.0.CO;2-M

[23] de Waal Malefyt R (2009) Interleukin-17 kick-starts T helper 1 cell differentiation. Immunity 31: 700–702. 19932069 10.1016/j.immuni.2009.11.002

[24] Mosmann TR, Sad S (1996) The expanding universe of T-cell subsets: Th1, Th2 and more. Immunol Today 17: 138–146. 8820272 10.1016/0167-5699(96)80606-2

[25] Nimmerjahn F, Ravetch JV (2005) Divergent immunoglobulin g subclass activity through selective Fc receptor binding. Science 310: 1510–1512. 16322460 10.1126/science.1118948

[26] Kang SJ, Locksley RM (2009) The inflammasome and alum-mediated adjuvanticity. F1000 Biol Rep 1: 15. 20948671 10.3410/B1-15 PMC2920669

[27] Montomoli E, Piccirella S, Khadang B, Mennitto E, Camerini R, et al (2011) Current adjuvants and new perspectives in vaccine formulation. Expert Rev Vaccines 10: 1053–1061. 21806399 10.1586/erv.11.48

[28] Muranski P, Restifo NP (2013) Essentials of Th17 cell commitment and plasticity. Blood 121: 2402–2414. 23325835 10.1182/blood-2012-09-378653 PMC3612853

[29] Burchill MA, Nardelli DT, England DM, DeCoster DJ, Christopherson JA, et al (2003) Inhibition of interleukin-17 prevents the development of arthritis in vaccinated mice challenged with Borrelia burgdorferi. Infect Immun 71: 3437–3442. 12761128 10.1128/IAI.71.6.3437-3442.2003 PMC155727

[30] Rueckert C, Guzman CA (2012) Vaccines: from empirical development to rational design. PLoS Pathog 8: e1003001. 23144616 10.1371/journal.ppat.1003001 PMC3493475

