# Correction: Cyclic GMP-AMP Displays Mucosal Adjuvant Activity in Mice

**DOI:** 10.1371/journal.pone.0123605

**Published:** 2015-03-31

**Authors:** The PLOS ONE Staff

The last author’s name is spelled incorrectly. The correct name is: Christine Rueckert. The correct citation is: Škrnjug I, Guzmán CA, Rueckert C (2014) Cyclic GMP-AMP Displays Mucosal Adjuvant Activity in Mice. PLoS ONE 9(10): e110150. doi:10.1371/journal.pone.0110150

There is an error in the first sentence of the “Spleen cell proliferation assay” subsection of the Materials and Methods. The correct sentence is: Spleen cells (4×10^5^) were stimulated with 40 μg/ml OVA, incubated for 72 h at 37°C, then incubated for another 16 h in the presence of 20 μCi/ml ^3^H-thymidine (PerkinElmer, USA) and harvested on Filtermat A filters (PerkinElmer, USA) using cell harvester (Inotech, Switzerland).
