# Supplementary figures and images for: Cyclic GMP-AMP Displays Mucosal Adjuvant Activity in Mice
Source: PLoS One. 2014 Oct 8;9(10):e110150. doi: 10.1371/journal.pone.0110150 (PMC4190368; doi:10.1371/journal.pone.0110150)

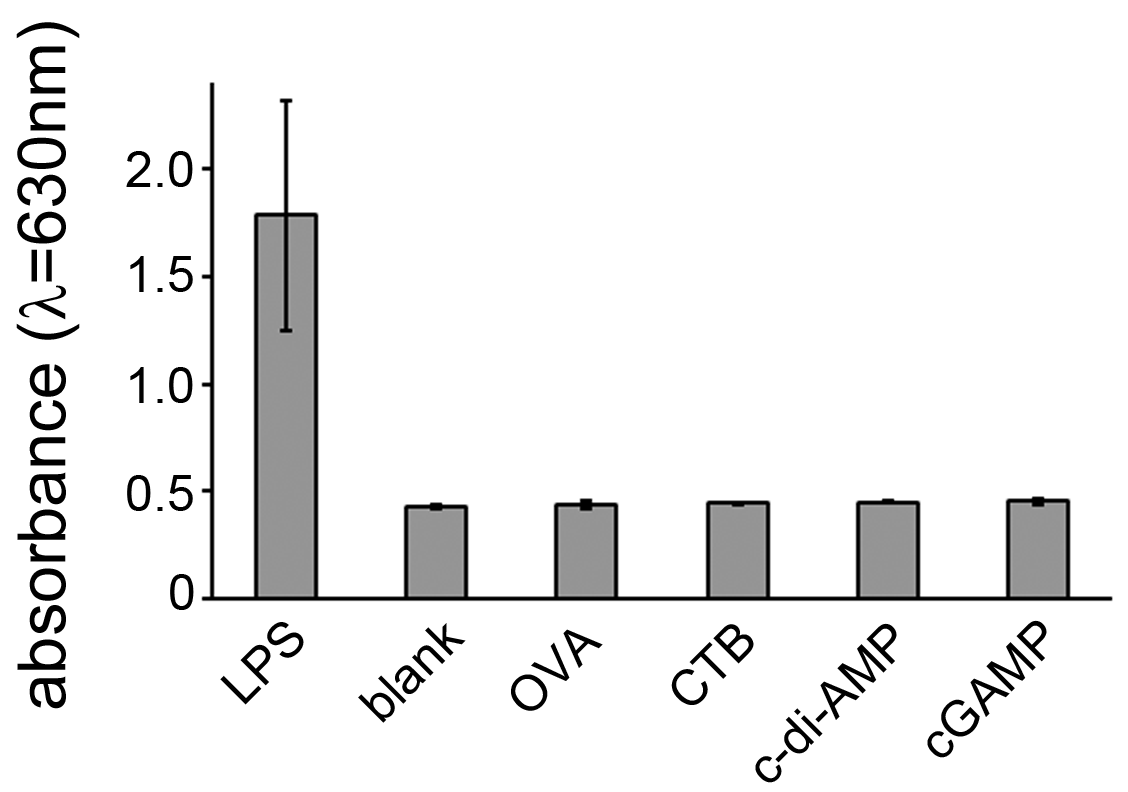

Supplement: Figure S1 — Test of reagents used in immunization experiments for endotoxin activity. Potential LPS contamination of the model antigen OVA and the adjuvants CTB, c-di-AMP and cGAMP was tested by employing the HEK-Blue LPS Detection Kit (Invivogen, USA) according to the manufacturer’s instructions. The substances were applied in the medium of the HEK-Blue cultures at the following concentrations: LPS with 1.625 ng/ml, OVA with 15 µg/ml, CTB with 25 µg/ml, c-di-AMP and cGAMP with 25 µg/ml. LPS was clearly detected by the readout absorbance whereas the absorbance values of OVA, CTB, c-di-AMP and cGAMP are at the same level as the blank value. The values represent duplicates, the error bars are SD. (TIF) [file pone.0110150.s001.tif]

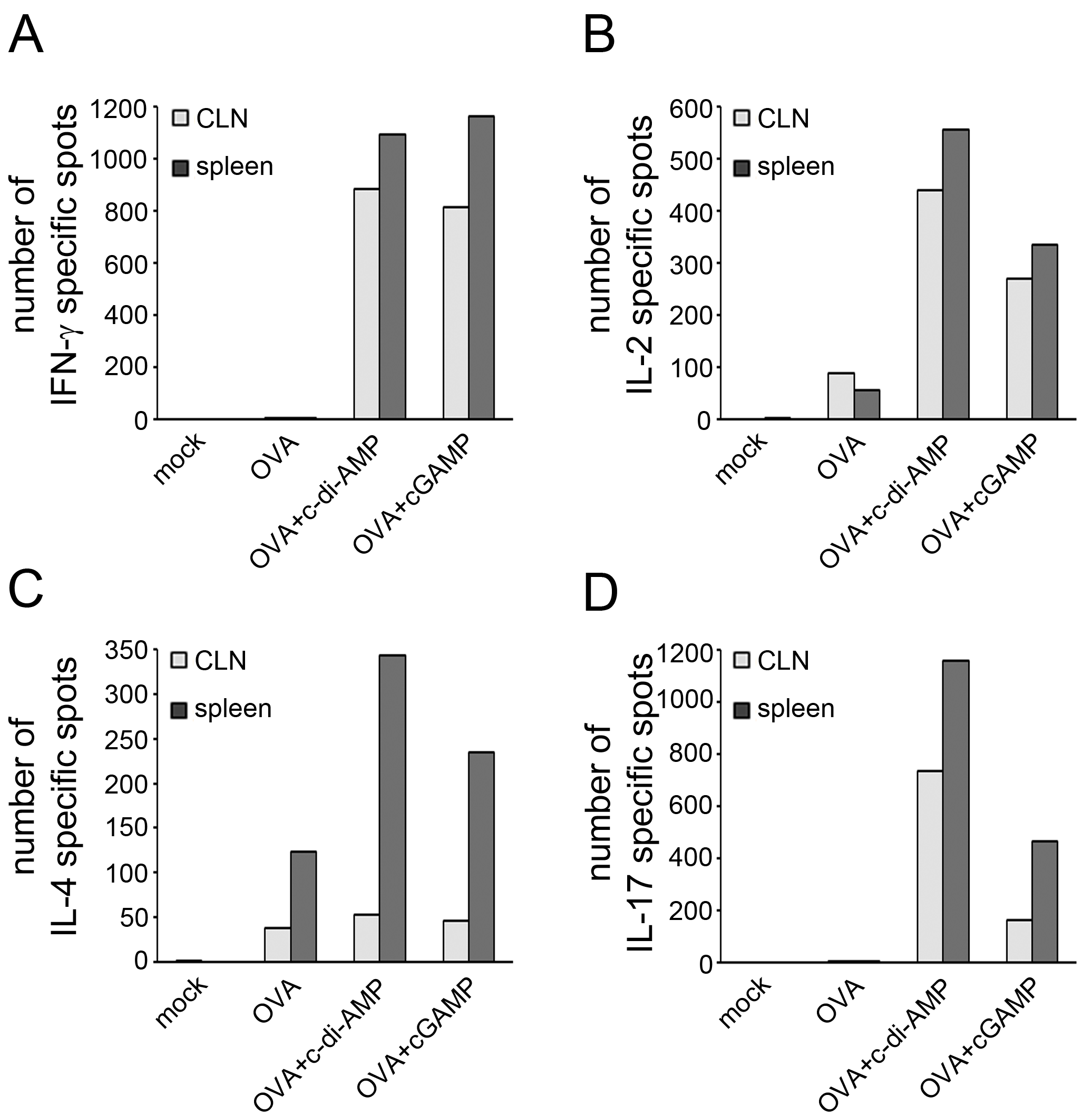

Supplement: Figure S2 — cGAMP-promotes antigen-specific cytokine production by cells of the spleen and the cervical lymph nodes (CLN). Mice were immunized with OVA alone or OVA adjuvanted with either c-di-AMP or cGAMP. Cells from the spleen and the CLN of these mice were re-stimulated with OVA and analyzed for the production of the cytokines (A) IFN-γ, (B) IL-2, (C) IL-4 and (D) IL-17 in ELISPOT assays. The number of spots is given for 106 cells. (TIF) [file pone.0110150.s002.tif]

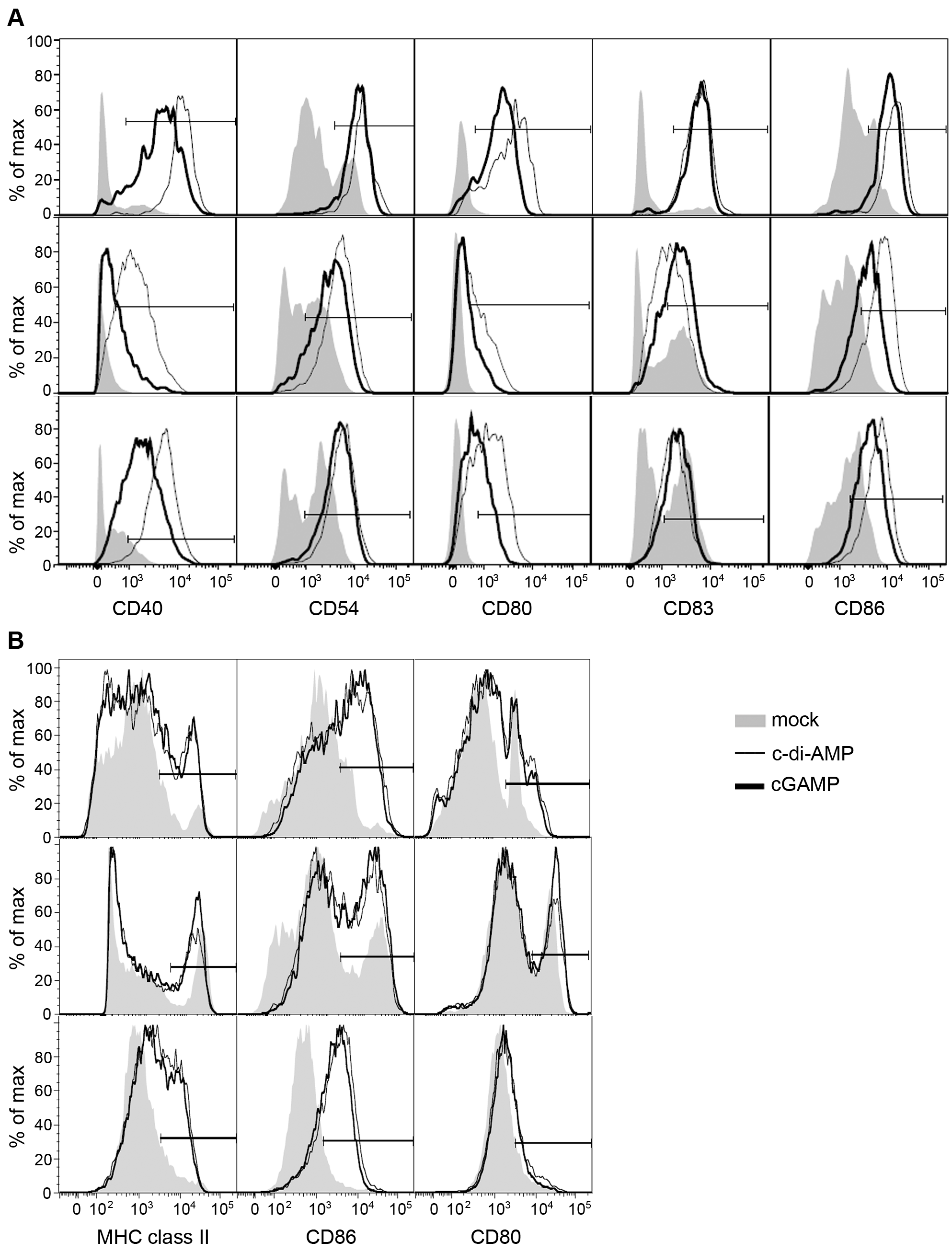

Supplement: Figure S3 — cGAMP up-regulates the surface expression of activation markers on human and murine dendritic cells. Human PBMC-derived DCs (A) and murine bone marrow-derived DCs (B) were stimulated in vitro with c-di-AMP, cGAMP or left untreated (mock) for 24 h. The DCs were decorated with fluorophore-conjugated antibodies against the markers CD40, CD54, CD80, CD83, CD86 and MHC class II (I-Ab) and further analyzed by flow cytometry. Histograms for activation marker analysis on CD11c+ cells are shown for each single experiment, one row representing data of one experiment. The horizontal bars show the applied gates for marker-positive cells. (TIF) [file pone.0110150.s003.tif]

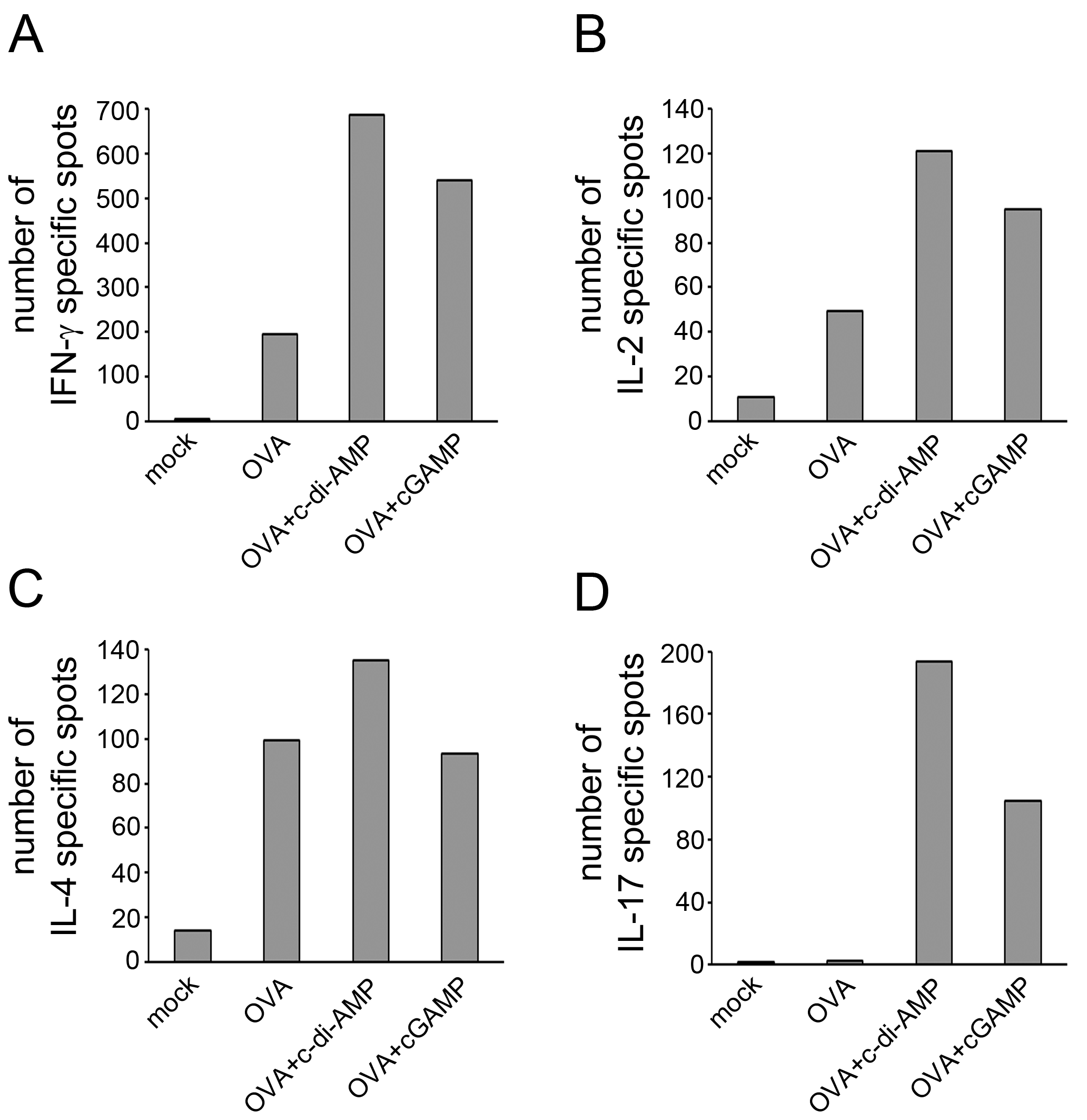

Supplement: Figure S4 — cGAMP-promoted antigen-specific cytokine production by spleen cells persists for six weeks after the last immunization boost. Mice were immunized with OVA alone or OVA adjuvanted with c-di-AMP or cGAMP. The mice were sacrificed six weeks after the last boost and spleen cells of these mice were re-stimulated with OVA and analyzed for the production of the cytokines (A) IFN-γ, (B) IL-2, (C) IL-4 and (D) IL-17 in ELISPOT assays. The number of spots is given for 106 cells. (TIF) [file pone.0110150.s004.tif]
